# Human biomonitoring data interpretation and ethics; obstacles or surmountable challenges?

**DOI:** 10.1186/1476-069X-7-S1-S13

**Published:** 2008-06-05

**Authors:** Ovnair Sepai, Clare Collier, Birgit Van Tongelen, Ludwine Casteleyn

**Affiliations:** 1 Chemical hazards and Poisons Division, Health Protection Agency, Chilton OX11 0RQ, UK; 2 AEA Technology plc, Harwell International Business Centre, Didcot OX11 0QR, UK; 3 European Commission, DG Environment, Unit B3 – Biotechnology, Pesticides & Health, Avenue de Beaulieu 9, Brussels Belgium; 4 Center for Human Genetics, Herestraat 49, B – 3000 Leuven, Brussels, Belgium

## Abstract

The use of human samples to assess environmental exposure and uptake of chemicals is more than an analytical exercise and requires consideration of the utility and interpretation of data as well as due consideration of ethical issues. These aspects are inextricably linked.

In 2004 the EC expressed its commitment to the development of a harmonised approach to human biomonitoring (HBM) by including an action in the EU Environment and Health Strategy to develop a *Human Biomonitoring Pilot Study*. This further underlined the need for interpretation strategies as well as guidance on ethical issues. A workshop held in December 2006 brought together stakeholders from academia, policy makers as well as non-governmental organisations and chemical industry associations to a two day workshop built a mutual understanding of the issues in an open and frank discussion forum. This paper describes the discussion and recommendations from the workshop.

The workshop developed key recommendations for a Pan-European HBM Study:

1. A strategy for the interpretation of human biomonitoring data should be developed.

2. The pilot study should include the development of a strategy to integrate health data and environmental monitoring with human biomonitoring data at national and international levels.

3. Communication strategies should be developed when designing the study and evolve as the study continues.

4. Early communication with stakeholders is essential to achieve maximum efficacy of policy developments and facilitate subsequent monitoring.

5. Member states will have to apply individually for project approval from their National Research Ethics Committees.

6. The study population needs to have sufficient information on the way data will be gathered, interpreted and disseminated and how samples will be stored and used in the future (if biobanking) before they can give informed consent.

7. The participants must be given the option of anonymity. This has an impact on follow-up.

8. The pilot study should develop guidelines and best practice for Ethics for pan European studies.

In conclusion all participants felt there that there has to be stakeholder involvement in any planned pan-European Human Biomonitoring Study and the format of the workshop was appropriate for such dialogue.

## Introduction

Biomonitoring is a very useful exposure assessment tool to assess uptake of chemicals. It has been extensively applied in the occupational health setting and has in recent years received increasing attention as a means to accurately measure low levels of environmental chemicals in human tissue. The use of human tissue to assess environmental exposures is more than an analytical exercise and requires consideration of the utility and interpretation of the data as well as due consideration of the ethical issues. These two aspects are inextricably linked.

In 2003 the European Commission (EC) announced its Environment and Health Strategy. This strategy contained many aspects of research and development addressing key priorities in the developing field of environmental public health. This led to the European Union (EU) Environment and Health Action Plan and included an action to develop a coherent approach to human biomonitoring. The EC, supported by a multidisciplinary working group of Member States representatives (Implementation Group on Human Biomonitoring) and by an Expert team to Support BIOmonitoring (ESBIO) is preparing an EU Pilot Project. The Pilot study has been delayed and it is hoped it will be launched by the end of 2008.

This approach would build on the current capabilities in many member states (MS) and not only address analytical and procedural differences between scientific institutes across Europe that lead to a loss of comparability of results, but also address legislation and policy differences between MS. The aims of such a pilot study include the development of strategies for data interpretation and communication to the study participants as well as to numerous other stakeholders. These elements are inextricable from the issues around obtaining approval from National Research Ethics Committees. These aims are also further compounded by the needs of a multinational (pan European) study.

On behalf of the EC (DG ENV), AEA Technology plc and the UK Health Protection Agency organised a stakeholder workshop to address issues surrounding data interpretation and ethical considerations of Human Biomonitoring (HBM). The organisation was in close cooperation with European Centre for Ecotoxicology & Toxicology of Chemicals (ECETOC) and the Health & Environment Alliance (HEAL, formerly EPHA Environment Network) and the meeting was hosted by the Ministry of the Flemish Community, Department of Environment, Nature & Energy in Brussels on the 6^th ^and 7^th ^December 2006.

This workshop brought together stakeholders from across Europe with expertise and interests in human biomonitoring. The participants included academics, policy makers, and representatives from Non-Governmental Organization and chemical industry associations. The purpose of this paper is to summarise the discussion and recommendations from the workshop and to present some of the current wider reaching issues. One of the main aims of the workshop was to produce recommendations for the pilot project and ESBIO.

## Data interpretation issues

The aims of the pilot study should be transparent and realistic: it should build capacity across Europe; harmonise methods and improve efficiency; develop best practice across Europe; provide data that can be compared cross boundary; link with other environment and health research projects; develop policy relevant research recommendations; and ultimately produce a framework for environment and health research across Europe.

Advances in analytical chemistry have led to the detection of an ever-increasing number of chemicals with reduced limits of detection in human samples. This has produced a dilemma of how to interpret the data and understand health impacts of the exposures detected and ultimately how to communicate this information to the public at individual and societal levels. Recently there has been a number of reviews, workshops, committees and publications proposing frameworks for the interpretation of human biomonitoring data [1-4]. The development of a standard strategy to interpret human biomonitoring data would be a huge advance in the field. In order to develop a standard framework it is important to understand the type and quality of data required to interpret the results.

The EU emphasizes the development of a coherent approach to human biomonitoring, to assure appropriate risk assessment and management for chemicals that influence human health. Biological monitoring data without appropriate health based limits can be used to determine trends or develop reference ranges (background exposure values). However, it is not possible to determine risk of adverse health effects or predict such adverse health effects using human biomonitoring data alone, unless biological guidance values have been determined for the chemicals of concern. This has been done for a few chemicals such as lead and cadmium. The integration of health and exposure data is the ultimate tool in public health. The ultimate goal of a pan European Environment and Human Health research programme would be to collect human health, environmental exposure, and biomonitoring data in a fully integrated system.

## Science to policy

Although many HBM studies are carried out across the EU, few of them have the specific aim to underpin the development of policy or even to directly involve policy makers in the planning implementation and final communication strategy. In 1977, Council Directive on Biological Screening of the Population for Lead (77/312/EEC) committed the EU member states to apply a common procedure for biological screening in order to assess the exposure of the population to lead outside the working environment [5]. Currently the World Health Organisation (WHO) coordinates the fourth in a series of surveys on determining the level of persistent organic pollutants (POPs), such as PCBs in human breast milk through out the world. The aim is to test the efficiency of policy agreements under the Stockholm Convention on POPs. There are many other examples – for European level policy relevant studies to be carried out it is necessary to consult policy representatives from all MS. Furthermore, to be able to monitor the impact of such policy there is a need to harmonize data gathering and surveillance.

Communication is a dynamic task and requires the development of tailored material for many groups of stakeholders. Early and regular two-way communication with all stakeholders may provide a means to assess the utility and ensure the appropriate direction of the program of work. The use of workshops to discuss key issues is a useful means of not only instigating discussion but also leads to trust and 'buy in' from the participants.

## Specific issues and recommendations

1. A harmonised European human biomonitoring programme is necessary and timely.

2. The aims of the pilot study should be transparent and realistic.

3. A sound strategy for the interpretation of human biomonitoring data should be developed.

4. The pilot study should include the development of a strategy to integrate health data and environmental monitoring data with human biomonitoring data at a national and international level.

5. Communication strategies should be in place when designing the study and develop as the study continues.

6. Early communication with key stakeholders is essential in order to achieve maximum efficacy of any policy developments and facilitate subsequent monitoring.

## Ethical issues

Research should be undertaken in accordance with commonly agreed standards of good practice such as are laid down in the Declaration of Helsinki. These fundamental and widely accepted ethical principles, largely derived from medical practice, are:

• Beneficence – 'do positive good'

• Non-maleficance – 'do no harm'

• Informed Consent

• Privacy and dignity

There are a number of key phrases that describe the system of ethical standards that the contemporary social and medical research establishment has created to try to protect better the rights of their research participants. The principle of voluntary participation requires that people are not coerced into participating in research. However, the participants should be reimbursed in line with the inconvenience they incur. The offer of incentives such as 'cinema tickets' or a voucher is practiced in some MS and may need to be considered for the pan European study. Closely related to the notion of voluntary participation is the requirement of informed consent. Essentially, this means that prospective research participants must be fully informed about the procedures and risks involved in the research and must give their consent to participate. Ethical standards also require that researchers will not put participants in a situation where they might be at risk of harm as a result of their participation. All MS must meet these standards and any communication plan should include information on the interpretation of the data produced.

Almost all research guarantees the participant's confidentiality – the participants are assured that identifying information will not be made available to anyone who is not directly involved in the study. For the purposed of a pan European study the data within country should be coded before it is collated on European database and national data protection legislation must be adhered to.

The stricter standard is the principle of anonymity. This essentially means that the participant will remain anonymous throughout the study – even to the researchers themselves. Clearly, the anonymity standard is a stronger guarantee of privacy and must be adhered to if a participant wishes not to know their individual results in any form. This has implications for a European Environment and Health Strategy where HBM data and environmental data are to be integrated. It will be necessary to make sure the data are geographically identifiable if not linked to individual participants. This will also impact on the pan-European HBM study if sequential samples from individuals or interventions are foreseen. This is a major consideration when designing the study.

In 1998 the European Group on Ethics (EGE) stated that there was an urgent need to regulate human tissues in the EU market. A series of meetings led to the drafting of the Tissue and Cells Directive (TCD, Directive 2004/23/EC) [6] that has now been adopted. However, national level legislation may differ substantially and will need to be considered. For example, in the UK there is a Human Tissue Act 2004 which stipulates where and how samples can be stored and requires licences to be applied for; it also provides guidelines on consent.

As the European biomonitoring study is an exposure and uptake assessment study and not a clinical trial, the added complications with the ethical issue of a person's right to service need be considered in the context of public health interventions. The need for service normally encompasses treatment or use of a placebo. In the context of a programme of monitoring for involuntary environmental exposure the need for service considers exposure mitigation in circumstances where high exposures are detected. The possibility of repeat sampling and/or interventions to reduce exposure has further impact on the participants 'right not to know'.

It is clear that there needs to be a procedure that assures that researchers will consider all relevant ethical issues in formulating the research design. All Member MSs have Research Ethics Committees (RECs) and although most of the legislation has some commonality as discussed above there are some conflicting issues. It is important that any research effort on a pan European scale must comply with National guidelines and protocols and it will be necessary for each MS to seek ethical approval on an individual basis.

There is a general recognition that the ethics environment is changing and international generated guidelines [7] and standards, such as those developed by the European Union, are one of several influences on this process of change. Research is increasingly international in scope in terms of topic, and data generated. New international research networks funded by programmes such as those of the European Union (e.g. the FP6 programme) result in greater opportunity for collaborative research across borders [8]. Currently it is necessary for each participating Member State to obtain ethical approval individually. The ideal situation would be that international projects be able to apply for 'international ethical approval'. This would require EU level Ethic Committees with adequate representation or knowledge of each member states' requirements. Subsequent to the initial pan European pilot study it may be possible to develop European Guidelines for Ethics in Human Biomonitoring in particular. This would be a significant output from the pilot study.

The starting premise of any new framework must be to review the current practice as is being carried out but the EU funded projects such as RESPEPT [9], ESBIO, NewGeneris [10], ECNIS [10] and others. In the UK, for example, the Economic and Social Research Council (ESRC) has produced a Research Ethics Framework [11] that has been developed after informed understanding of global and other regional or national legal and regulatory frameworks. This framework clearly sets out the issues and principles as were also discussed in the stakeholder workshop. The framework goes further to develop protocols and checklists. There are many such developments that require some streamlining. An output from the pilot study would be generic guidelines and templates to guide subsequent EU research projects. This was one of the recommendations from the workshop.

## Specific issues and recommendations

1. Member states will have to apply for project approval from their National Research Ethics Committees.

2. The study population needs to have sufficient information on the way data will be gathered, interpreted and disseminated and how samples will be stored and used in the future (if biobanking) before they can give informed consent.

3. The participants must be given the option of anonymity. This has an impact on follow-up.

4. The pilot study should develop guidelines and best practice for future pan European studies. This may lead to developing an EU level Research Ethics Committee.

## Conclusion

The application of human biomonitoring in public health studies needs the involvement of all stakeholders as part of the discussion group at the planning stage of a project. This will allow research to focus on issues related to public concern and be policy relevant.

## Competing interests

The authors declare that they have no competing interests.
